# Influence of Immunocastration and Diet on Meat and Fat Quality of Heavy Female and Male Pigs

**DOI:** 10.3390/ani11123355

**Published:** 2021-11-24

**Authors:** Leticia Pérez-Ciria, Francisco Javier Miana-Mena, María Carmen López-Mendoza, Javier Álvarez-Rodríguez, Maria Angeles Latorre

**Affiliations:** 1 Departamento de Producción Animal y Ciencia de los Alimentos, Instituto Agroalimentario de Aragón-IA2, Universidad de Zaragoza-CITA, C/Miguel Servet 177, 50013 Zaragoza, Spain; malatorr@unizar.es; 2 Departamento de Farmacología y Fisiología, Instituto Agroalimentario de Aragón-IA2, Universidad de Zaragoza-CITA, C/Miguel Servet 177, 50013 Zaragoza, Spain; jmiana@unizar.es; 3 Departamento de Producción Animal y Ciencia y Tecnología de los Alimentos, Universidad Cardenal Herrera-CEU, C/Tirant lo Blanc 7, Alfara del Patriarca, 46115 Valencia, Spain; clopez@uchceu.es; 4 Departamento de Ciencia Animal, Universidad de Lleida, Av. Alcalde Rovira Roure 191, 25198 Lleida, Spain; javier.alvarez@udl.cat

**Keywords:** immunocastration, high energy, low protein, meat quality, fat quality, pig

## Abstract

**Simple Summary:**

Sufficient fat cover is necessary for an optimum dry-curing process of Teruel dry-cured hams. However, in recent years, gilts intended for this type of hams are characterized by lack of fat deposition, since male pigs are surgically castrated, to miss boar taint, and castration increases fatness. Thus, immunocastration or the increase of energy in the diet or the decrease of dietary crude protein and amino acids could solve this problem. On the other hand, the surgical castration of male pigs could be banned in the near future in the European Union. Hence, immunocastration could be a solution, as well. However, immunocastrated males seems to present lower fatness than surgically castrated males. Thus, it would be interesting to study feeding plans that increase fatness. Therefore, two experiments were conducted, one with females and another with males, to evaluate the effect of immunocastration and diet on meat and fat quality. In conclusion, immunocastration is a good practice in gilts, as it improves meat quality and technological fat quality. However, in the case of males, this strategy deteriorates meat quality when compared with surgical castration, and it should be accompanied with a high-energy diet or a low-crude-protein diet to guarantee an adequate fat consistency.

**Abstract:**

Two experiments were carried out; one with female pigs and the other with male pigs destined for Teruel dry-cured ham production, to evaluate the effect of immunocastration (entire gilts-EG vs. immunocastrated gilts-IG and surgically castrated males vs. immunocastrated males-IM) and diet (control vs. high energy vs. low crude protein and amino acids) on meat quality and fat composition. Fifteen meat samples and eight fat samples of each treatment were analyzed in both experiments. In the case of males, six fat samples per treatment were analyzed to determine boar taint. Immunocastration is a good strategy in gilts intended for dry-cured ham production because improves meat composition; however, in males, immunocastration impairs the results of pork chemical composition compared with surgical castration. The IG presented a lower polyunsaturated/saturated fatty acids ratio than EG, improving fat technological quality. Diets had little effect on pork or fat quality in gilts, but a high-energy level using oilseeds and a low-crude-protein and -amino-acids diet from 80 to 137 kg of body weight could be interesting in IM to maintain or increase fat consistency, respectively. Moreover, in general, immunocastration is effective in avoiding boar taint in males.

## 1. Introduction

Currently, a significant percentage of pig carcasses intended for the Protected Designation of Origin (PDO) Teruel dry-cured ham are declared unfit. The main cause is the lack of fat covering the ham [1], which favors the salting process and prevents the excessive drying of the cured pieces [2]. Likewise, trained panelists have detected modest intramuscular fat (IMF) content in the meat of these animals [3], a parameter which is positively related to juiciness and negatively to hardness [4]. These problems appear mainly in females [1,5], since males destined for this PDO have to be castrated, to avoid boar taint, and castration increases the accretion of fat tissue [6]. The usual castration carried out in male piglets in Spain is surgical. The higher carcass fatness generated by this type of castration has been also observed in females [7]. Feeding strategies could resolve, in part, these problems. The rise of dietary energy level [8] or the reductions in crude protein (CP) and amino acids (AA) content [9] seem to increase fat cover at the *gluteus medius* muscle (GM), although the results on the IMF content are not so conclusive. The immunocastration (immunization against gonadotrophin releasing factor-GnRF) of females could be another option for increasing their fatness, because the surgical castration of female pigs reared under intensive conditions is banned in the European Union [10]. On the other hand, in male pigs, for welfare reasons, immunocastration is also emerging as a possible alternative to surgical castration. However, immunocastrated males (IM) seem to present lower fat deposition than surgically castrated males (SCM) [11], which would be undesirable for dry-cured ham production. In this context, it would also be reasonable to study the feeding strategies of these animals in order to optimize their fatness content and composition. Lastly, it should be noted that immunocastrated gilts (IG) and IM could be expected to have different feeding patterns than entire gilts (EG) and SCM, respectively, and thus it would be important to evaluate appropriate feeding plans for them that optimize the quality of the final product. Therefore, two trials were carried out, one with female pigs and the other with male pigs, all of them destined for the PDO Teruel ham, with the aim of assessing the effect of immunocastration and diet on meat and fat quality.

## 2. Materials and Methods

Two trials were carried out and they are described as follows. Pigs were raised in compliance with the Spanish Policy for Animal Protection [12]. All the experimental procedures used followed the requisites of the Ethical Committee of the University of Zaragoza (ref. PI29/18).

### 2.1. Pig Husbandry and Experimental Design

In trial 1, a total of 192 Duroc × (Landrace × Large White) gilts of 40.3 ± 4.80 kg body weight (BW) (84 ± 3 days of age) were used. Half of them were immunocastrated using two doses of Vacsincel^®^ (Zoetis Spain S.L., Alcobendas, Madrid, Spain): the first dose at 58.1 ± 6.39 kg of BW (102 ± 3 days of age) and the second dose at 77.0 ± 8.12 kg of BW (122 ± 3 days of age). The other half were EG throughout the trial.

In trial 2, a total of 144 Duroc × (Landrace × Large White) male pigs of 35.3 ± 4.10 kg BW (78 ± 3 days of age) were used. Half of them were surgically castrated during the first week of life and the other half were immunocastrated using three doses of Improvac^®^ (Zoetis Belgium SA, Louvain-la-Neuve, Belgium): the first dose at the end of post-weaning period, at approximately 25 kg of BW (56 ± 3 days of age), as required by the Teruel ham *Consortium*. Immunocastrated pigs were immunized against GnRF for the second time at 57.7 ± 5.60 kg of BW (101 ± 3 days of age) and for the third time at 79.2 ± 7.20 kg of BW (122 ± 3 days of age), to ensure the effect of the immunization.

In both trials, upon arrival at the farm (Foz-Calanda, Teruel, Spain), animals were housed in groups of eight in pens of 9 m^2^. Three experimental diets were offered to all of them during the grower and the finisher periods: (i) a control diet, with a nutritional profile similar to the recommendations of FEDNA [13] for this type of animal; (ii) a diet with a greater energy content than the control diet, but maintaining similar CP and AA percentages; and (iii) a diet with lower CP and AA contents than the control diet, but of similar energy level. In all diets, the ideal protein content was maintained [13] and the change between the grower and the finisher feeds was carried out on a fixed day. The grower diets were supplied from 122 to 149 ± 3 days of age (approximately 78–106 kg of BW) and the finisher diets from 150 ± 3 days of age to the day before slaughter (approximately 106–136 kg of BW). The ingredients, estimated nutrient composition and analyzed nutrient composition of the experimental diets are shown in Table 1, Table 2 and Table 3, respectively. Feed, in pellet form, and water were provided *ad libitum*.

Therefore, in both trials there were six experimental treatments; two types of gilts (EG vs. IG) or two types of males (SCM vs. IM) × three diets (control vs. high-energy vs. low-CP and -AA). 

### 2.2. Feed Analyses

The determinations of gross energy, dry matter, ash, starch, ether extract, neutral detergent fiber, CP and total AA of the diets are detailed in Pérez-Ciria et al. [14]. Fatty acids were extracted and quantified following the one-step procedure described by Sukhija and Palmquist [15] with minor modifications. Each sample (250 mg) in the presence of toluene (1 mL containing 10 mg/mL of the internal standard–C15:0 and another milliliter of toluene) and acetyl chloride in methanol (3 mL at 1/10) was shaken 30 s at a low speed and later heated for 2 h at 70 °C in a shaking water bath. Then, each sample was cooled to room temperature and 5 mL of potassium carbonate were added. Subsequently, each sample was vortexed for 30 s at a high speed and centrifuged for 5 min at 3500 rpm, and the upper phase was taken and 1 g of anhydrous sodium sulfate was added. Each sample was vortexed and centrifuged again. Finally, the upper phase was collected to identify and quantify fatty acid methyl esters as described in López-Bote et al. [16] using a gas chromatograph (HP 6890 Series GC System) with a flame ionization detector and a capillary column (HP-Innowax: 30 m × 0.32 mm × 0.25 µm cross-linked polyethylene glycol).

### 2.3. Slaughtering and Meat and Fat Sampling

In both trials, slaughter took place when animals achieved 134 and 137 kg of BW on average, for females and males, respectively (between 178 and 199 days old). The day before slaughter, pigs were not fed for 5 h and were moved to a commercial slaughterhouse (Teruel, Spain), where they were kept in lairage for 10 h without feed but with *ad libitum* access to water. Animals were stunned in a CO_2_ atmosphere, exsanguinated, scalded, dehaired, singed, eviscerated and split lengthwise. After refrigeration at 2 °C (1 m/s air speed; 90% relative humidity) for 5 h, the carcasses were processed according to commercial standards.

In each trial, a total of 90 carcasses (15 per treatment) were chosen at random in order to study their meat and fat quality. From each one, a piece of 100 ± 10 g of the left *longissimus thoracis* muscle (LT) and other similar piece of the left GM were taken. Samples of the LT were used to analyze thawing and cooking losses, color parameters and maximum stress and those of the GM were utilized to determine chemical composition. Additionally, from 48 left-side hams (eight per treatment) randomly chosen, a piece of 100 ± 10 g of the subcutaneous fat (including skin, fat layers and lean) was sampled to analyze the fatty acid profile. In addition, in the trial of males, 36 subcutaneous fat samples (six per treatment) were intended for determining the compounds responsible for boar taint. The subcutaneous fat samples of IM were taken from pigs with a testicular width (both testicles) shorter than 11 cm. That criterion was implemented in Brazil, a country where the use of immunocastration is widespread, for acceptance at the slaughterhouses of IM, based on the reduced risk of boar taint [17]. Additionally, five subcutaneous fat samples of IM that showed a testicular width greater than 11 cm were also intended to be analyzed for boar taint (to be used as positive control samples). All samples (meats and fats) were vacuum-packaged in individual plastic bags and preserved at −20 °C for subsequent analyses.

### 2.4. Meat Quality Traits

Firstly, the LT samples were thawed for 24 h at 4 °C, removed from plastic bags, blotted dry for 15 min and weighed. Thawing losses were calculated considering the fresh and thawed weight. Afterwards, color was measured with a spectrophotometer (CM-2600d, Konica Minolta Holdings, Inc., Osaka, Japan) in CIELAB space [18], with an Illuminant D65 and an observer angle of 10°, previously calibrated according to manufacturer recommendations. The mean of two random measures in each sample was used to obtain lightness (L*), redness (a*), yellowness (b*), chroma $\left(C_{ab}^{*}\right)$ and hue-angle (*h_ab_*) values. Later, in those samples, cooking losses were determined by the method described by Honikel [19]. The samples were weighed, placed in individual plastic bags and cooked in a water bath (Precisterm, J.P. Selecta S.A., Barcelona, Spain) at 75 °C to reach a core temperature of 70 °C. During the cooking, the internal temperature was monitored through a thermocouple type T connected to a data logger (testo 177-T4, Testo GmbH, Lenzkirch, Germany). Then, the cooked samples were cooled, blotted dry and weighed again. For the cooking-losses calculation, pre- and post-cooking weights were considered. Then, maximum stress was measured, following the procedure for the Warner–Bratzler shear test described by Honikel [19]. The cooked samples were cut in prism-shaped pieces with a 100 mm^2^ (10 × 10 mm) cross-section with the fiber direction parallel to a long dimension of at least 30 mm. Five prisms per sample were sheared at right angles to the fiber axis using a Warner–Bratzler device, with a cross-head speed of 2.5 mm/s, attached to an Instron universal testing machine (Model 5543, Instron Ltd., Buckinghamshire, UK), itself attached to a computer. Maximum stress was defined as the load at maximum peak shear force per unit of cross-section [20].

Chemical composition (moisture, protein and IMF) was determined according to the procedures of Boletín Oficial del Estado [21]. Firstly, the GM samples were thawed for 24 h at 4 °C and minced. Moisture was analyzed using an oven (Memmert UFE500, Schwabach, Germany) over 48 h at 102 °C, protein with a 2300 Kjeltec Analyzer Unit (Foss Tecator, Höganäs, Sweden) and IMF by an ANKOM^XT15^ Extration System (ANKOM Techonology, Macedon, NY, USA) after the samples had been hydrolyzed by an ANKOM^HCL^ Hydrolysis System.

### 2.5. Fatty Acid Profile of the Subcutaneous Fat

Firstly, the fat samples spent, individually, 1 min in a microwave at 350 W. Afterward, from each sample, 30 μL of melted fat were taken and 1 mL of hexane and 1 mL of methylated mixture (69% methanol, 29% toluene and 2% sulfuric acid by volume) were added. Samples were placed in the oven at 70–80 °C for 2 h and shaken manually every 15 min. Later, they were cooled and fatty acid methyl esters were recovered from the upper phase, separated and quantified by gas chromatography, as described for the feeds. The percentages of total saturated fatty acids (SFA), monounsaturated fatty acids (MUFA) and polyunsaturated fatty acids (PUFA), and also the PUFA/SFA ratio, total n-3 and n-6 percentages and the n-6/n-3 ratio were calculated from individual fatty acid proportions.

### 2.6. Boar Taint Compounds

Androstenone, skatole and indole concentrations in the subcutaneous fat samples were analyzed according to the procedures of Hansen-Møller [22], Pauly et al. [23] and Batorek et al. [24], with little modification. Firstly, samples were left at room temperature for 30 min. Then, 10 to 20 g of each, without lean and skin, were cut in cubes and liquefied in a microwave for 2 × 2 min at 350 W. The liquefied fat was removed and centrifuged for 20 min at 10,800× *g* at 40 °C and kept at 50 °C. Then, 0.5 ± 0.01 g of supernatant was placed in 2.5-mL Eppendorf tubes and internal standards were added (1 mL of methanol containing 0.5 mg/L of androstanone and 0.05 mg/L of 2-methylindole). After stirring for 30 s, each tube was incubated for 5 min at 30 °C in an ultrasonic water bath (FB 15061, Fisher Scientific, Illkirch Cedez, France), kept on ice for 20 min and then centrifuged for 20 min at 10,800× *g* at 4 °C. Finally, supernatant was transferred with a syringe of 1 mL into a high-performance liquid chromatography (HPLC) vial for androstenone, skatole and indole analysis with an HPLC system from Agilent Technologies (1200 series) as described by Batorek et al. [24]. Concentrations were expressed as µg/g of liquid fat. The detection limits were 0.20 µg/g for androstenone and 0.03 µg/g for skatole and indole. For data analysis, when the concentrations of skatole or indole were below the limit of detection, they were defined as half of the limit of detection.

### 2.7. Statistical Analyses

In both trials, data were analyzed as a randomized factorial design (2 × 3) using the GLM procedure of the Statistical Analysis System (SAS 9.4 software, SAS Institute Inc., Cary, NC, USA). In trial 1, the model included the type of gilt (EG and IG) and diet (control, high-energy and low-CP and -AA) as main effects. In trial 2, the model included the type of castration (surgical and immunological) and diet (control, high-energy and low-CP and -AA) as main effects. In both trials, interactions (type of gilt or type of castration × diet) were included in the models for parameters that were significant (*p* < 0.05) and excluded from the final models when they were not significant. Least square means were separated using the PDIFF option.

Residuals’ normality was verified with Shapiro–Wilk test using the UNIVARIATE procedure. When normality was not achieved, variables were transformed with $\sqrt{x}$ or 1/x or $x^{2},$ if it was possible. In cases in which normality could not be found with data transformation, variables were analyzed using non-parametric methods: Mann–Whitney U test was carried out when types of gilt or types of castration were compared and Kruskall–Wallis test when different diets were compared.

The pig was the experimental unit (per treatment: *n* = 15 for meat quality, *n* = 8 for fatty acid profile and *n* = 6 for boar taint compounds). A *p*-value < 0.05 was considered as a significant difference and between 0.05 and 0.10 as a tendency.

## 3. Results and Discussion

### 3.1. Trial 1: Meat Quality and Fat Composition in Female Pigs

**Figure 1:**
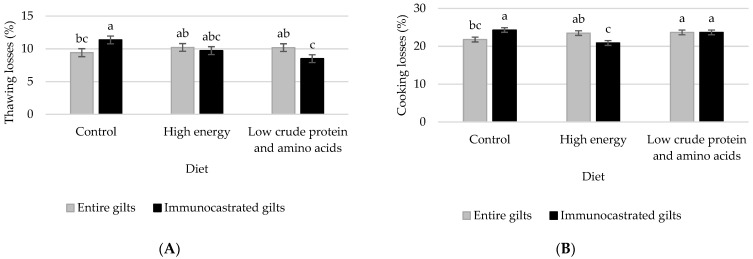
Significant interactions (*p* < 0.05) between type of gilt and diet on water holding capacity indicators (**A**): thawing losses and (**B**): cooking losses of the *longissimus thoracis* muscle. Diets during the grower period: control (2.33 Mcal net energy-NE/kg, 16% crude protein-CP and 0.77% standardized ileal digestible lysine-SID Lys), high energy (2.48 Mcal NE/kg, 16% CP and 0.77% SID Lys) and low CP and amino acids (AA) (2.33 Mcal NE/kg, 14% CP and 0.67% SID Lys). Diets during the finisher period: control (2.33 Mcal NE/kg, 14.5% CP and 0.63% SID Lys), high energy (2.48 Mcal NE/kg, 14.5% CP and 0.63% SID Lys) and low CP and AA (2.33 Mcal NE/kg, 12.5% CP and 0.54% SID Lys). Different letters (a, b, c) denote significant differences between least square means (*p* < 0.05).

The only significant interactions type of gilt × diet were observed for thawing and cooking losses (Figure 1). Whereas, with the control diet, IG had greater thawing losses than EG, with the high-energy diet there were no differences, and with the low-CP and -AA diet IG presented lower thawing losses than EG (*p* = 0.011). In addition, whereas with the control diet IG had greater cooking losses than EG, with the high-energy diet the opposite effect was observed, and with the low-CP and -AA diet there was no difference (*p* = 0.0005). Therefore, when immunocastration is practiced in gilts, the increase of energy or decreased CP and AA in the feed could generate a reduction in cooking or thawing losses. Some authors [25,26,27] observed similar cooking losses when EG and IG were fed a standard diet, but it is known that factors relative to the freezing and cooking conditions have great influence in the water losses of meat.

The effect of gilt immunocastration and diet on meat quality is shown in Table 4. There was no influence of immunocastration of gilts (*p* > 0.10) on any meat-color trait, in agreement with a great deal of reports [25,28,29]. Maximum stress was also similar (*p* = 0.887) in the loins of both types of gilts. Bohrer et al. [25], Martinez-Macipe et al. [30] and Xue et al. [31] also found no differences between EG and IG in other texture parameters, i.e., shear force. Meat from IG had similar protein proportion (*p* = 0.204) but lower (*p* = 0.034) moisture percentage than that from EG. Bohrer et al. [25] and Pérez-Ciria et al. [27] detected this effect on moisture only numerically. Also, meat from IG presented greater (*p* = 0.018) IMF proportion than that from EG, which could have a positive effect on texture and appearance of dry-cured hams and could reduce ham’s weight losses during the dry-curing process [2,4]. There is some unanimity that gilt immunocastration seems to increase IMF percentage [26,28], although some authors [27,29] have detected this effect only numerically (not significantly), which may be due to the use of a small number of animals or to the fact that the breed used was very fatty, mitigating the effect. In fact, in these last studies [27,29], fat thickness at GM muscle was significantly higher in IM than in EG.

Dietary treatments had no significant effect (*p* > 0.10) on the color or texture of the meat, corroborating the findings of several works [8,32,33]. In addition, pork chemical composition (moisture, protein and IMF) was not affected (*p* > 0.10) by feeding strategies, in agreement with the results of Suarez-Belloch et al. [8] by increasing energy levels from 2280 to 2420 kcal NE/kg and Rodríguez-Sánchez et al. [33] by reducing CP and lysine (Lys) contents from 14.5% CP and 0.71% total Lys to 14% CP and 0.59% total Lys. However, this could depend on the type of diet evaluated and on the length of the period tested. Suárez-Belloch et al. [32], applying a greater dietary CP and Lys restriction (from 17.2% CP and 0.77% Lys to 10.6% CP and 0.42% Lys) from 90 to 130 kg of BW, observed that meat protein content decreased. In addition, this last study [32] next to the work of Teye et al. [34], in which CP and Lys contents were reduced from 21% and 1.0% to 18% and 0.7%, respectively, from 40 to 100 kg of BW, detected that CP and Lys reduction increased IMF percentage.

The effect of gilt immunocastration and diet on subcutaneous fat composition is provided in Table 5. 

Total SFA proportion was greater (*p* = 0.003) in IG than in EG, owing to the higher C16:0 (*p* = 0.051) and C18:0 (*p* = 0.001) contents. The percentage of total MUFA was not affected (*p* = 0.771) by female immunocastration, mainly because the major MUFA (C18:1n-9) was similar (*p* = 0.968) between EG and IG. Total PUFA proportion was lower (*p* = 0.006) in IG, due to the lower C18:2n-6 (*p* = 0.007), C18:3n-3 (*p* = 0.017), C18:3n-6 (*p* = 0.015), C18:4n-3 (*p* = 0.024), C20:3n-6 (*p* = 0.054) and C20:4n-6 (*p* = 0.010) contents. Therefore, PUFA/SFA ratio (*p* = 0.004) and total n-3 (*p* = 0.007) and n-6 (*p* = 0.006) percentages were lower in IG than in EG. It is worth noting that the value of PUFA/SFA ratio obtained in both groups was nutritionally acceptable (the target is ≥0.4) [35]. Some authors [27,28] have obtained results similar to ours. However, others [29,36] did not detect significant influence of female immunocastration on fat quality, which could be due to the use of a different crossbreed [29] or the smaller interval between the second dose of immunocastration and the slaughter [36]. As was mentioned previously, there is certain unanimity in the literature about the greater fatness in IG than in EG [25,26,28]. In general, the larger the fat deposits, the higher the proportion of fatty acids from de novo synthesis (SFA and MUFA) and the lower the percentage of PUFA (provided only by dietary lipids) stored in adipose tissue, since PUFA are diluted [37,38]. The higher proportion of SFA and the lower of PUFA found in IG would imply that these gilts would present more firm and cohesive fat, being better for meat technological processes, but less healthy [39]. In addition, the pork pieces of IG would have better storage stability and flavor, due to a lower susceptibility to oxidative spoilage [39].

Dietary treatments only had significant influence on some MUFA. Fat from gilts fed the low-CP and -AA diet presented higher (*p* = 0.026) percentage of C16:1n-7 than that from gilts fed the high-energy diet, placing those gilts fed the control diet in an intermediate position. Animals that ate the low-CP and -AA diet or the control diet had greater (*p* = 0.013) C18:1n-7 proportion than those fed the high-energy diet, and gilts fed the alternative diets to control showed lower (*p* = 0.005) percentage of C20:1n-9 than those fed the control diet. Hence, the increase in dietary energy or the decrease in CP and AA of the diet had no effect (*p* > 0.10) on total SFA, MUFA and PUFA contents, PUFA/SFA ratio, total n-3 and n-6 proportions and n-6/n-3 ratio. Suarez-Belloch et al. [8] and Rodríguez-Sánchez et al. [33] found similar results to those reported in the current manuscript after increasing dietary energy or decreasing CP and Lys in the diet, respectively. Although in the current study there were no differences in IMF content between feeding strategies, the differences in minor MUFA may be related with dietary-induced modifications in the Stearoyl–CoA desaturase (SCD) gene-family expression, which in turn may contribute to porcine adipocyte differentiation and adipogenesis [40].

### 3.2. Trial 2: Meat Quality, Fat Composition and Boar Taint Compounds in Male Pigs

The effect of the type of castration and diet on meat quality of male pigs is shown in Table 6. The type of male castration had no impact (*p* > 0.10) on thawing or cooking losses, which agrees with the findings of Pauly et al. [41] and Seiquer et al. [42]. However, other authors [24,43,44] detected that pork from IM had higher cooking losses than that from SCM, justifying this with the increase in protein oxidation in the case of IM, reducing the ability of the muscle to bind water. Meat from IM presented lower (*p* = 0.027) L* and tended to show lower (*p* = 0.084) *h_ab_* than that from SCM, which are parameters strictly linked with human perception of pork color [45], whereas a*, b* and $C_{ab}^{*}$ were similar in both groups (*p* > 0.10). In the literature, the effect of the type of castration on color traits is not unanimous. Some reports [41,46,47] did not find differences between SCM and IM in any color trait but others [48,49,50] showed, as in the present work, that meat from IM presented lower L* value. Daza et al. [36], Andreo et al. [50] and Seiquer et al. [42] observed that IM presented lower a* and $C_{ab}^{*}$ values, and Škrlep et al. [44] observed the opposite effect. Different genetic types, pre-slaughter handling and conditions of data collection could contribute to pork color [51], generating differences unrelated to animal husbandry.

The type of castration had no influence (*p* = 0.233) on meat texture, evaluated as maximum stress, which confirms the results of other works [24,26,44]. It is worth noting that, when trained panelists have evaluated pork from SCM vs. pork from IM, they have established that IM presents similar tenderness to SCM [30,47]. Regarding the chemical composition of muscle, there was no influence of the type of castration on protein content (*p* = 0.856), but meat from IM presented greater (*p* = 0.004) moisture percentage and lower (*p* = 0.001) IMF percentage than that from SCM. The results observed about IMF proportion between groups are consistent with works of several authors [30,42,48]. The low IMF percentage generated by immunocastration in male pigs would be not desirable because it could have a negative influence on some texture and appearance attributes of dry-cured hams [4]. The reason for such difference between groups would be associated to IM pigs behaving as entire males (EM) until the second dose of immunocastration, and EM (and also IM until the second dose injection) presenting higher levels of testosterone than SCM [52], castrated from the first week of life, which would decrease fat mass [53]. Nevertheless, some authors [24,44,54] did not detect this difference as significant and this might be due to the different genetics used, the age at which immunocastration doses were administered and the time elapsed between the second vaccination and slaughter. Thereby, shortening the time from the second vaccination to slaughter may allow the expression of more differences in IMF accretion between IM and SCM.

The tested diets had no influence (*p* = 0.459) on thawing losses, but they had an impact (*p* = 0.002) on cooking losses. Meat from males fed the low-CP and -AA diet presented greater cooking losses than that from males fed the control or the high-energy diet, without observing differences between these last two diets. The higher weight loss during cooking with the CP and AA restriction could impair juiciness [55], being a negative aspect for pork quality. Matthews et al. [56] also found no effect of increasing dietary energy on thawing and cooking losses, and Sirtori et al. [57] observed that animals restrictively fed the lowest CP and AA diet had higher cooking losses as well. The experimental feeds had no significant impact (*p* > 0.05) on any color trait, in agreement with Matthews et al. [56] by increasing dietary energy level, and with Suárez-Belloch et al. [32] and Tejeda et al. [58] by reducing CP and Lys contents. Likewise, meat tenderness was not affected (*p* = 0.332) by dietary treatments. Reducing CP and AA contents, Millet et al. [59] and Suárez-Belloch et al. [32] did not observe an influence on shear force, either. Therefore, the potential mild negative effects of reduced CP and AA diets on cooking losses may be counterbalanced by similar meat tenderness and a potential reduction in feeding costs. As in the case of gilts (Trial 1, Table 4), and although a higher IMF content in meat was expected by decreasing CP and AA and/or by increasing energy in diets, finally meat chemical composition was similar (*p* > 0.10), irrespective of feeding strategy. Some authors [33,59] also observed no effect when reducing dietary protein or Lys content, but others [32,57] did, mainly in IMF percentage, and it was probably because the restriction of those nutrients was more severe. In the case of dietary energy level, Matthews et al. [56] also detected no influence when increasing energy level in diets, but Liu et al. [60] achieved a higher IMF proportion, probably due to the difference in energy between diets was more pronounced.

Regarding fat composition, some significant interactions between type of castration and diet were detected (Table 7).

The most notable interactions were the following. The high-energy diet affected similarly on total PUFA and total n-6 percentages and PUFA/SFA ratio, irrespective of the type of castration, but IM had higher values thereof with the control diet and lower with the low-CP and -AA diet than SCM (*p* < 0.05). Also, the fat from both types of males had similar content in total SFA percentage when control or high-energy diets were given, but IM presented higher content thereof than SCM with the low-CP and -AA diet (*p* < 0.04). In addition, SCM and IM fed the high-energy diet or the low-CP and -AA diet showed similar total n-3 proportion, whereas IM fed the control diet had higher percentage of total n-3 than SCM fed this diet (*p* = 0.004). Therefore, in the case of IM, the use of a standard diet would lead to a decrease in the consistency of their fat, whereas a low-CP and -AA diet would improve it, although their fat could be less healthy, and with a high-energy diet the fat consistency would be maintained similarly to that of SCM.

The effect of the type of castration and diet, as main effects, on the remaining results of subcutaneous fat composition of male pigs is presented in Table 8. The fat from IM presented lower (*p* = 0.041) total MUFA percentage than that from SCM, owing to the lower (*p* = 0.028) C18:1n-9 content. The reason for it would be in line with the preceding discussion of Trial 1; backfat thickness is generally thinner in IM [11], and the less developed the backfat, the less is the proportion of MUFA stored in the adipose tissue arising from de novo synthesis [37]. Mackay et al. [61] and Asmus et al. [62] also found lower total MUFA proportion in the fat of IM, whereas others authors [41,63] failed to detect differences between IM and SCM in this parameter. The lower MUFA content found in IM would be positive from a technological point of view, since MUFA have a negative influence on firmness and cohesiveness of fat tissue [64]. On the other hand, this finding would be less desirable from a health point of view, because MUFA have a beneficial effect on coronary heart disease risk [65]. Besides, IM had similar (*p* = 0.920) n-6/n-3 ratio than SCM, in agreement with the findings of Font-i-Furnols et al. [54] and Daza et al. [36].

In respect of experimental diets, fat from males fed the low-CP and -AA diet had lower (*p* = 0.0005) C15:0 percentage than that from males fed the control or the high-energy diets. Pigs that received the alternative diets to control presented lower percentages of C17:0 (*p* = 0.002) and C17:1 (*p* < 0.0001). Additionally, males fed the high-energy diet presented lower (*p* = 0.009) C18:1n-7 content and tended to show greater (*p* = 0.066) C18:1n-9 percentage than those fed the control or the low-CP and -AA diet. This tendency may be promoted by the higher content of C18:1n-9 in high-energy feed, which was supplemented with greater levels of palm oil. Pigs that received the alternative diets presented a higher (*p* = 0.024) n-6/n-3 ratio, which is not desirable from a health-focused point of view [66]. Madeira et al. [67] also found that CP and Lys reduction increased n-6/n-3 ratio. On the other hand, no influence (*p* = 0.403) of feeding strategy was observed on total MUFA content, which agrees with the works of Suárez-Belloch et al. [32] and Tejeda et al. [58].

Table 9 shows the influence of type of castration in male pigs and diet on the boar-taint compounds analyzed in the samples of subcutaneous fat from SCM and IM whose testicular width was less than 11 cm. No significant interactions (*p* > 0.05) between type of castration and diet were found, and therefore, the results are shown as main effects. The concentration of androstenone was below the detection limit in all cases (0.20 µg/g). Likewise, skatole and indole concentrations were not influenced (*p* > 0.10) by the type of castration and the contents of both were low (<0.09 µg/g). Thus, SCM and IM had androstenone and skatole levels below the thresholds values for sensory acceptance (0.5–1.0 µg/g and 0.20–0.25 µg/g, respectively) [68]. These findings agree with those published by other authors [24,41,69]. However, Weiler et al. [70], injecting the second dose of immunocastration closer to slaughter, reported that IM had higher levels of androstenone and indole than SCM, although their mean values were equally low (<0.11 µg/g). It has been seen that immunization against GnRF hinders the formation of testicular steroids, including androstenone, by blocking hypothalamic-pituitary-gonadal axis, and therefore prevents the accumulation of this compound in fat tissue [71,72]. Besides, deprivation of testicular steroids, especially 17β-estradiol and androstenone, seems to reduce skatole formation in the intestine and to accelerate its degradation in the liver, and consequently would lead to decrease skatole accumulation in fat [72]. In addition, a similar effect could occur in the case of indole accumulation [69].

Regarding the fat samples of IM that showed a testicular width greater than 11 cm (data not shown), one of them had an androstenone concentration lower than the detection limit (0.20 µg/g) and the remaining presented values between 0.49 and 1.73 µg/g. The values of skatole ranged from 0.08 to 0.42 µg/g and those of indole from 0.06 to 0.23 µg/g. In the current trial, the proportion of IM that exceeded the target testicular width was 11%. Therefore, not all of these IM seem to have high values in all boar-taint compounds, but some of them did. Several reports [54,73,74] have also found a little proportion (between 0.74 and 11.8%) of IM designated as “non-responders”. The reasons could be that those pigs are missed at vaccination moments in group-housing systems, or respond poorly to the immunization against GnRF, or have health problems, malnutrition or stress [48,71,75]. Therefore, disregarding the non-responders, immunocastration was effective in the prevention of boar taint.

No significant influence (*p* > 0.05) of the diet was observed on boar-taint compounds, although it should be noted that male pigs fed alternative diets to the control, especially those that received the low-CP and -AA diet, tended (*p* = 0.059) to present lower levels of skatole. This compound is a product of bacterial degradation of the amino acid tryptophan in the large intestine [71]. With the alternative diets, especially with the low-CP and -AA diet, pigs ingested a lower percentage of tryptophan, and therefore it could be expected to obtain a lower skatole concentration. However, it should be noted that tryptophan is mainly absorbed in the small intestine, and consequently is only available to a limited degree for microbial degradation in the large intestine, as the gut mucosa cell debris are a major source of tryptophan [76,77,78]. In the studies of Westergaard and Mortensen (cited by Malmfors et al. [79]) and Nold et al. [80], feed-protein content had no significant impact on skatole concentration. In the literature, the effect of increasing energy in the diet on skatole concentration is controversial. Lundström et al. [77] observed that providing a diet with high-nutrient density (high energy), skatole concentration decreased, while Neupert et al. [81] and Westergaard and Mortensen (cited by Malmfors et al. [79]) found the opposite effect. Lundström et al. [77] explained that the high-nutrient-density diet had a lesser content of fibre, which will decrease the fermentative process in the large intestine, reducing the number of bacteria and thus the microbial protein that could act as an extra source of tryptophan. However, Claus and Raab [82] described that a rise in energy supply leads to an increase in insulin-like growth factor-1 (IGF-1), rising the degree of mitosis in the intestine, and thus a parallel increase of the apoptotic cells provides the substrate for skatole formation. In the case of androstenone, nutritional influences are attributed to energy or to specific compounds in the ration, although the effect of nutrition, here, is less important than for skatole [83]. Claus et al. [76] found higher androstenone levels with a greater increase in energy content than that of the current trial, maybe because the high-energy diet increases IGF-1, which would stimulate Leydig cell steroidogenic responsiveness, and thus, androstenone formation [84]. Nevertheless, Zeng et al. [85], with a smaller difference of energy between the high- and low-energy diets, did not observe any effect of feed energy content on androstenone concentration, as in the current study. Further research should be carried out to better understand the effect of dietary energy or AA levels on boar taint compounds.

## 4. Conclusions

Gilt immunocastration increases IMF content and generates a more adequate fatty acid profile for the curing process, although it also could result in less healthy products. In female pigs, irrespective of whether they are entire or immunocastrated, a high-energy diet or a low-CP and -AA diet at 76 to 134 kg of BW has little influence on meat quality and fat composition. Immunocastration in male pigs deteriorates meat chemical composition compared with surgical castration, because IMF is decreased, but it provides a similar or a better fatty acid profile, in terms of fat consistency, when a high-energy diet or a low-CP and -AA diet, respectively, is given at 80 to 137 kg of BW. However, a low-CP and -AA diet in IM could produce less healthy fat. In general, male pig immunocastration reduces boar-taint compounds as much as surgical castration, although it has to be noted that some animals are “non-responders”, implying higher levels of those compounds in their fat.

## Figures and Tables

**Table 1:** Ingredients of the tested diets (%, as-fed basis).

Ingredient	Grower Diet (78 to 106 kg Body Weight)			Finisher Diet (106 to 136 kg Body Weight)		
	Control	High Energy	Low CP and AA	Control	High Energy	Low CP and AA
Corn	35.0	33.9	35.0	35.0	32.5	35.0
Wheat	18.0	18.0	18.4	17.0	18.0	18.1
Barley	17.6	15.0	21.0	21.1	21.8	25.0
Oat	9.00	8.72	11.0	11.0	8.00	12.0
Soybean meal 47% CP	17.8	18.7	11.9	13.6	14.4	7.69
Palm oil	0.53	3.65	0.34	0.36	3.36	0.08
Calcium carbonate	0.79	0.78	0.80	0.85	0.85	0.86
Sodium chloride	0.45	0.45	0.45	0.45	0.45	0.45
Monocalcium phospate	0.26	0.27	0.31	0.13	0.13	0.18
L-Lysine 50%	0.23	0.21	0.30	0.14	0.12	0.23
L-Threonine	0.02	0.02	0.02	-	-	0.01
DL-Methionine	0.02	0.02	0.01	-	-	-
L-Tryptophan	-	-	-	0.01	0.01	0.01
Premix ^1^	0.40	0.40	0.40	0.40	0.40	0.40

CP: crude protein; AA: amino acids. ^1^ The following were provided per kilogram of complete diet: 6.5 IU vitamin A; 1.5 IU vitamin D3; 15 mg α-tocopherol; 3 mg vitamin B2; 1 mg vitamin B6; 0.02 mg vitamin B12; 15 mg nicotinic acid; 8 mg pantothenic acid; 100 mg choline chloride; 100 mg Zn (ZnO); 50 mg Mn (MnO); 250 mg Fe (FeCO_3_); 10 mg Cu (CuSO_4_·5H_2_O); 0.2 mg Se (Na_2_O_3_Se); 2 mg BHT; 1 mg I (KI); 500 FYT 6-phytase.

**Table 2:** Estimated nutrient composition of the tested diets (%, as-fed basis).

Nutrient	Grower Diet (78 to 106 kg Body Weight)			Finisher Diet (106 to 136 kg Body Weight)		
	Control	High Energy	Low CP and AA	Control	High Energy	Low CP and AA
Net energy, Mcal/kg	2.33	2.48	2.33	2.33	2.48	2.33
CP	16.0	16.0	14.0	14.5	14.5	12.5
Digestible AA						
Lysine	0.77	0.77	0.67	0.63	0.63	0.54
Methionine	0.24	0.24	0.21	0.21	0.20	0.18
Methionine + Cysteine	0.49	0.49	0.44	0.44	0.43	0.39
Threonine	0.50	0.50	0.43	0.43	0.43	0.36
Tryptophan	0.16	0.16	0.14	0.15	0.15	0.13

CP: crude protein; AA: amino acids.

**Table 3:** Analyzed nutrient content of the tested diets (%, as-fed basis).

Nutrient	Grower Diet (78 to 106 kg Body Weight)			Finisher Diet (106 to 136 kg Body Weight)		
	Control	High Energy	Low CP and AA	Control	High Energy	Low CP and AA
Gross energy, Mcal/kg	3.99	4.12	3.92	3.91	4.12	3.95
Dry matter	88.7	88.2	88.0	88.0	89.4	88.1
Ash	4.18	4.19	4.17	3.85	3.98	3.65
Starch	42.1	40.3	44.0	44.5	47.8	49.0
Ether extract	3.55	5.88	3.44	3.00	5.65	3.73
Neutral detergent fiber	10.9	10.2	10.5	10.5	8.96	10.2
CP	16.2	15.9	14.4	14.5	15.1	12.7
Total AA						
Lysine	0.98	0.98	0.79	0.76	0.77	0.71
Methionine	0.28	0.27	0.25	0.24	0.25	0.23
Threonine	0.62	0.60	0.59	0.56	0.58	0.51
FA, % of total FA						
C12:0	3.45	1.66	3.35	3.26	1.72	2.50
C14:0	0.58	0.65	0.41	0.34	0.84	0.83
C16:0	23.3	30.2	25.5	25.0	34.6	27.9
C16:1n-7	0.18	0.19	0.20	0.18	0.17	0.20
C16:1n-9	0.05	0.05	0.06	0.06	0.04	0.06
C18:0	3.20	3.61	3.18	3.18	4.05	3.49
C18:1n-7	0.70	0.61	0.73	0.71	0.62	0.65
C18:1n-9	30.9	33.6	31.1	31.6	34.2	32.9
C18:2n-6	34.9	27.4	32.8	32.6	21.6	28.8
C18:3n-3	1.89	1.46	1.66	1.62	1.05	1.29
C18:4n-3	0.24	0.12	0.28	0.43	0.32	0.44
C20:0	0.16	0.07	0.18	0.38	0.34	0.40
C20:1n-9	0.46	0.32	0.50	0.61	0.40	0.57

CP: crude protein; AA: amino acids; FA: fatty acids.

**Table 4:** Effect of immunocastration and diet on the meat quality of gilts.

Trait	Type of Gilt		SEM ^1^ (*n* = 45)	Diet ^2^			SEM ^1^ (*n* = 30)	*p*-Value ^3^	
	Entire	Immunocastrated		Control	High Energy	Low CP and AA		Gilt	Diet
Color traits ^4^									
Lightness, L*	35.0	35.0	0.69	35.2	34.8	35.0	0.85	0.982	0.936
Redness, a*	2.58	3.13	0.279	2.83	2.70	3.02	0.342	0.168	0.793
Yellowness, b*	14.8	15.1	0.27	15.1	14.8	15.0	0.32	0.389	0.784
Chroma, $C_{ab}^{*}$	15.1	15.5	0.27	15.4	15.1	15.4	0.33	0.281	0.722
Hue angle, *h_ab_*	80.2	78.4	1.06	79.5	79.7	78.7	1.30	0.216	0.860
Maximum stress ^4^, N/cm^2^	40.9	41.2	1.61	40.4	42.8	40.0	1.97	0.887	0.566
Chemical composition ^5^, %									
Moisture	72.2	71.6	0.19	72.0	72.0	71.8	0.24	0.034	0.798
Protein	23.6	23.4	0.11	23.3	23.5	23.7	0.14	0.204	0.145
Intramuscular fat ^6^	2.70	3.57	0.239	3.29	3.18	2.93	0.293	0.018	0.494

^1^ SEM: standard error of the mean. ^2^ Grower period: control (2.33 Mcal net energy-NE/kg, 16% crude protein-CP and 0.77% standardized ileal digestible lysine-SID Lys), high energy (2.48 Mcal NE/kg, 16% CP and 0.77% SID Lys) and low CP and amino acids (AA) (2.33 Mcal NE/kg, 14% CP and 0.67% SID Lys). Finisher period: control (2.33 Mcal NE/kg, 14.5% CP and 0.63% SID Lys), high energy (2.48 Mcal NE/kg, 14.5% CP and 0.63% SID Lys) and low CP and AA (2.33 Mcal NE/kg, 12.5% CP and 0.54% SID Lys). ^3^ No significant interactions (type of gilt × diet) were found (*p* > 0.05). ^4^ Color and texture analyses were carried out with samples of the *longissimus thoracis* muscle. ^5^ Proximate composition analyses were carried out with samples of the *gluteus medius* muscle. ^6^ Least square means and SEM of the original data and *p*-values obtained with the transformed data.

**Table 5:** Impact of immunocastration and diet on subcutaneous fat composition (fatty acids expressed as % of total fatty acids, except in ratios) of gilts.

Trait	Type of Gilt		SEM ^1^ (*n* = 24)	Diet ^2^			SEM ^1^ (*n* = 16)	*p*-Value ^3^	
	Entire	Immunocastrated		Control	High Energy	Low CP and AA		Gilt	Diet
C14:0	1.13	1.16	0.023	1.17	1.10	1.16	0.028	0.327	0.154
C14:1 ^4^	0.013	0.012	0.0007	0.012	0.012	0.013	0.0009	0.434	0.738
C15:0	0.054	0.048	0.0023	0.050	0.055	0.048	0.0028	0.062	0.240
C15:1 ^4^	0.012	0.010	0.0008	0.011	0.013	0.010	0.0010	0.039	0.223
C16:0	22.0	22.6	0.22	22.2	22.2	22.6	0.27	0.051	0.478
C16:1n-7	1.57	1.55	0.042	1.53 ^ab^	1.47 ^b^	1.67 ^a^	0.051	0.654	0.026
C16:1n-9	0.337	0.294	0.0113	0.299	0.342	0.306	0.0138	0.011	0.067
C17:0	0.307	0.284	0.0142	0.301	0.295	0.290	0.0174	0.259	0.911
C17:1	0.271	0.243	0.0118	0.270	0.244	0.255	0.0145	0.105	0.449
C18:0	11.8	12.7	0.19	12.3	11.9	12.5	0.23	0.001	0.160
C18:1n-7	1.73	1.65	0.030	1.75 ^a^	1.60 ^b^	1.73 ^a^	0.036	0.063	0.013
C18:1n-9	42.8	42.8	0.25	42.8	43.3	42.3	0.31	0.968	0.092
C18:2n-6	15.6	14.3	0.34	14.9	15.2	14.8	0.41	0.007	0.777
C18:3n-3	0.728	0.670	0.0165	0.714	0.701	0.683	0.0202	0.017	0.556
C18:3n-6 ^5^	0.040 ± 0.017	0.031 ± 0.008	-	0.032 ± 0.006	0.040 ± 0.022	0.036 ± 0.009	-	0.015	0.236
C18:4n-3	0.058	0.049	0.0029	0.053	0.053	0.055	0.0036	0.024	0.873
C20:0 ^5^	0.248 ± 0.038	0.261 ± 0.031	-	0.267 ± 0.025	0.246 ± 0.047	0.251 ± 0.026	-	0.138	0.086
C20:1n-9	0.857	0.913	0.0202	0.956 ^a^	0.852 ^b^	0.848 ^b^	0.0247	0.057	0.005
C20:3n-6 ^4^	0.113	0.099	0.0049	0.110	0.107	0.101	0.0060	0.054	0.566
C20:4n-6	0.271	0.238	0.0086	0.258	0.253	0.252	0.0105	0.010	0.899
C20:5n-3 ^4^	0.033	0.031	0.0033	0.027	0.034	0.035	0.0041	0.549	0.273
Total SFA	35.5	37.1	0.36	36.3	35.8	36.8	0.44	0.003	0.233
Total MUFA	47.6	47.5	0.25	47.6	47.8	47.2	0.31	0.771	0.303
Total PUFA	16.9	15.4	0.36	16.1	16.4	16.0	0.44	0.006	0.807
PUFA/SFA	0.477	0.418	0.0136	0.445	0.462	0.437	0.0166	0.004	0.533
Total n-3	0.820	0.749	0.0173	0.793	0.787	0.773	0.0212	0.007	0.784
Total n-6	16.1	14.7	0.35	15.3	15.6	15.2	0.43	0.006	0.804
n-6/n-3	19.6	19.6	0.20	19.3	19.8	19.8	0.24	0.905	0.275

SFA: saturated fatty acids; MUFA: monounsaturated fatty acids; PUFA: polyunsaturated fatty acids. ^1^ SEM: standard error of the mean. ^2^ Grower period: control (2.33 Mcal net energy-NE/kg, 16% crude protein-CP and 0.77% standardized ileal digestible lysine-SID Lys), high energy (2.48 Mcal NE/kg, 16% CP and 0.77% SID Lys) and low CP and amino acids (AA) (2.33 Mcal NE/kg, 14% CP and 0.67% SID Lys). Finisher period: control (2.33 Mcal NE/kg, 14.5% CP and 0.63% SID Lys), high energy (2.48 Mcal NE/kg, 14.5% CP and 0.63% SID Lys) and low CP and AA (2.33 Mcal NE/kg, 12.5% CP and 0.54% SID Lys). ^3^ No significant interactions (type of gilt × diet) were found (*p* > 0.05). ^4^ Least square means and SEM of the original data and *p*-values obtained with the transformed data. ^5^ This variable has been analyzed using non-parametric methods and its data are presented as mean ± standard deviation. Within a row, least square means without a common letter (^a^, ^b^) differ (*p* < 0.05).

**Table 6:** Effect of type of castration and diet on meat quality of male pigs.

Trait	Type of Castration		SEM ^1^ (*n* = 45)	Diet ^2^			SEM ^1^ (*n* = 30)	*p*-Value ^3^	
	Surgical	Immunological		Control	High Energy	Low CP and AA		Castration	Diet
Thawing losses ^4, 5^, %	10.09	9.47	0.389	9.30	9.90	10.14	0.476	0.187	0.459
Cooking losses ^4, 5^, %	23.7	24.0	0.39	23.2 ^b^	23.2 ^b^	25.2 ^a^	0.48	0.491	0.002
Color traits ^5^									
Lightness, L*	34.8	32.2	0.82	33.6	33.4	33.5	1.01	0.027	0.990
Redness, a*	3.83	4.37	0.299	3.89	3.97	4.44	0.367	0.207	0.505
Yellowness, b*	14.8	14.2	0.33	14.5	14.3	14.6	0.40	0.182	0.817
Chroma, $C_{ab}^{*}$	15.4	14.9	0.31	15.1	15.0	15.4	0.38	0.302	0.775
Hue angle ^4^, *h_ab_*	75.5	72.5	1.22	74.9	73.9	73.2	1.49	0.084	0.695
Maximum stress ^4, 5^, N/cm^2^	40.4	37.6	1.91	38.0	41.3	37.7	2.33	0.233	0.332
Chemical composition ^6^, %									
Moisture	71.4	72.2	0.17	71.7	71.6	72.1	0.21	0.004	0.246
Protein	23.0	23.1	0.08	23.0	23.0	23.1	0.10	0.856	0.876
Intramuscular fat	4.44	3.40	0.220	4.02	4.15	3.58	0.269	0.001	0.293

^1^ SEM: standard error of the mean. ^2^ Grower period: control (2.33 Mcal net energy-NE/kg, 16% crude protein-CP and 0.77% standardized ileal digestible lysine-SID Lys), high energy (2.48 Mcal NE/kg, 16% CP and 0.77% SID Lys) and low CP and amino acids (AA) (2.33 Mcal NE/kg, 14% CP and 0.67% SID Lys). Finisher period: control (2.33 Mcal NE/kg, 14.5% CP and 0.63% SID Lys), high energy (2.48 Mcal NE/kg, 14.5% CP and 0.63% SID Lys) and low CP and AA (2.33 Mcal NE/kg, 12.5% CP and 0.54% SID Lys). ^3^ No significant interactions (type of castration × diet) were found (*p* > 0.05). ^4^ Least square means and SEM of the original data and *p*-values obtained with the transformed data. ^5^ Water losses, color and texture analyses were carried out with samples of the *longissimus thoracis* muscle. ^6^ Proximate composition analyses were carried out with samples of the *gluteus medius* muscle. Within a row, least square means without a common letter (^a^, ^b^) differ (*p* < 0.05).

**Table 7:** Interactions between type of castration and diet with regard to subcutaneous fat composition (fatty acids expressed as % of total fatty acids, except in ratios) of male pigs.

Diet ^1^	Control		High Energy		Low CP and AA		SEM ^2^ (*n* = 8)	*p*-Value
Type of Castration	Surgical	Immunological	Surgical	Immunological	Surgical	Immunological		
C16:0	23.3 ^abc^	22.6 ^c^	22.8 ^bc^	23.8 ^a^	22.7 ^bc^	23.5 ^ab^	0.31	0.019
C16:1n-9	0.259 ^b^	0.318 ^a^	0.290 ^ab^	0.277 ^ab^	0.296 ^ab^	0.268 ^b^	0.0150	0.012
C18:2n-6	13.8 ^bc^	15.7 ^a^	14.1 ^bc^	13.8 ^bc^	15.1 ^ab^	13.4 ^c^	0.47	0.002
C18:3n-3	0.660 ^bc^	0.754 ^a^	0.651 ^bc^	0.632 ^bc^	0.687 ^b^	0.617 ^c^	0.0232	0.003
C18:3n-6	0.025 ^c^	0.033 ^a^	0.032 ^ab^	0.030 ^abc^	0.028 ^abc^	0.026 ^bc^	0.0022	0.046
C18:4n-3	0.040 ^bc^	0.045 ^ab^	0.052 ^a^	0.034 ^c^	0.039 ^bc^	0.037 ^bc^	0.0039	0.010
C20:1n-9	0.967 ^a^	0.881 ^bc^	0.927 ^abc^	0.875 ^c^	0.874 ^c^	0.962 ^ab^	0.0351	0.042
C20:4n-6	0.211 ^b^	0.263 ^a^	0.230 ^ab^	0.226 ^b^	0.233 ^ab^	0.218 ^b^	0.0123	0.021
Total SFA	38.0 ^ab^	37.0 ^b^	37.1 ^b^	38.6 ^ab^	37.2 ^b^	39.0 ^a^	0.60	0.039
Total PUFA	14.9 ^bc^	16.9 ^a^	15.2 ^bc^	14.8 ^bc^	16.2 ^ab^	14.4 ^c^	0.50	0.002
PUFA/SFA	0.392 ^bc^	0.459 ^a^	0.411 ^abc^	0.384 ^c^	0.436 ^ab^	0.371 ^c^	0.0181	0.002
Total n-3	0.724 ^b^	0.829 ^a^	0.728 ^b^	0.691 ^b^	0.751 ^b^	0.682 ^b^	0.0259	0.004
Total n-6	14.1 ^bc^	16.1 ^a^	14.5 ^bc^	14.1 ^bc^	15.4 ^ab^	13.7 ^c^	0.48	0.001

SFA: saturated fatty acids; PUFA: polyunsaturated fatty acids. ^1^ Grower period: control (2.33 Mcal net energy-NE/kg, 16% crude protein-CP and 0.77% standardized ileal digestible lysine-SID Lys), high energy (2.48 Mcal NE/kg, 16% CP and 0.77% SID Lys) and low CP and amino acids (AA) (2.33 Mcal NE/kg, 14% CP and 0.67% SID Lys). Finisher period: control (2.33 Mcal NE/kg, 14.5% CP and 0.63% SID Lys), high energy (2.48 Mcal NE/kg, 14.5% CP and 0.63% SID Lys) and low CP and AA (2.33 Mcal NE/kg, 12.5% CP and 0.54% SID Lys). ^2^ SEM: standard error of the mean. Within a row, least square means without a common letter (^a–c^) differ (*p* < 0.05).

**Table 8:** Impact of type of castration and diet on subcutaneous fat composition (fatty acids expressed as % of total fatty acids, except in ratios) of male pigs.

	Type of Castration		SEM ^1^ (*n* = 24)	Diet ^2^			SEM ^1^ (*n* = 16)	*p*-Value ^3^	
	Surgical	Immunological		Control	High Energy	Low CP and AA		Castration	Diet
C14:0	1.22	1.20	0.022	1.22	1.18	1.23	0.027	0.664	0.488
C14:1	0.012	0.012	0.0006	0.012	0.012	0.011	0.0007	0.786	0.546
C15:0 ^4^	0.047	0.051	0.0021	0.055 ^a^	0.050 ^a^	0.041 ^b^	0.0025	0.195	0.0005
C15:1 ^4^	0.008	0.008	0.0003	0.009	0.008	0.007	0.0004	0.284	0.105
C16:1n-7 ^4^	1.59	1.61	0.045	1.66	1.54	1.60	0.055	0.648	0.266
C17:0	0.282	0.313	0.0118	0.342 ^a^	0.284 ^b^	0.267 ^b^	0.0145	0.064	0.002
C17:1	0.250	0.264	0.0097	0.308 ^a^	0.233 ^b^	0.231 ^b^	0.0118	0.329	<0.0001
C18:0	12.7	13.1	0.24	12.7	12.8	13.2	0.29	0.235	0.418
C18:1n-7 ^4^	1.70	1.67	0.034	1.76 ^a^	1.58 ^b^	1.73 ^a^	0.042	0.478	0.009
C18:1n-9	42.4	41.7	0.24	41.6	42.6	41.9	0.29	0.028	0.066
C20:0	0.263	0.252	0.0087	0.254	0.263	0.256	0.0106	0.407	0.809
C20:3n-6	0.096	0.097	0.0031	0.102	0.093	0.095	0.0039	0.768	0.235
C20:5n-3	0.025	0.027	0.0014	0.027	0.025	0.027	0.0018	0.219	0.688
Total MUFA	47.2	46.4	0.26	46.6	47.2	46.7	0.32	0.041	0.403
n-6/n-3	20.0	20.0	0.17	19.5 ^b^	20.2 ^a^	20.3 ^a^	0.21	0.920	0.024

MUFA: monounsaturated fatty acids. ^1^ SEM: standard error of the mean. ^2^ Grower period: control (2.33 Mcal net energy-NE/kg, 16% crude protein-CP and 0.77% standardized ileal digestible lysine-SID Lys), high energy (2.48 Mcal NE/kg, 16% CP and 0.77% SID Lys) and low CP and amino acids (AA) (2.33 Mcal NE/kg, 14% CP and 0.67% SID Lys). Finisher period: control (2.33 Mcal NE/kg, 14.5% CP and 0.63% SID Lys), high energy (2.48 Mcal NE/kg, 14.5% CP and 0.63% SID Lys) and low CP and AA (2.33 Mcal NE/kg, 12.5% CP and 0.54% SID Lys). ^3^ No significant interactions (type of castration × diet) were found (*p* > 0.05). ^4^ Least square means and SEM of the original data and *p*-values obtained with the transformed data. Within a row, least square means without a common letter (^a^, ^b^) differ (*p* < 0.05).

**Table 9:** Effect of type of castration and diet on boar-taint compounds (µg/g) in subcutaneous fat of male pigs.

	Type of Castration		Diet ^1^			*p*-Value ^2^	
	Surgical	Immunological	Control	High Energy	Low CP and AA	Castration	Diet
*n*	18	18	12	12	12		
Androstenone ^3^	bd	bd	bd	bd	bd	-	-
Skatole ^4^	0.028 ± 0.014	0.031 ± 0.018	0.037 ± 0.014	0.027 ± 0.019	0.025 ± 0.012	0.593	0.059
Indole ^4^	0.021 ± 0.011	0.027 ± 0.018	0.031 ± 0.019	0.018 ± 0.008	0.022 ± 0.013	0.267	0.149

^1^ Grower period: control (2.33 Mcal net energy-NE/kg, 16% crude protein-CP and 0.77% standardized ileal digestible lysine-SID Lys), high energy (2.48 Mcal NE/kg, 16% CP and 0.77% SID Lys) and low CP and amino acids (AA) (2.33 Mcal NE/kg, 14% CP and 0.67% SID Lys). Finisher period: control (2.33 Mcal NE/kg, 14.5% CP and 0.63% SID Lys), high energy (2.48 Mcal NE/kg, 14.5% CP and 0.63% SID Lys) and low CP and AA (2.33 Mcal NE/kg, 12.5% CP and 0.54% SID Lys). ^2^ No significant interactions (type of castration × diet) were found (*p* > 0.05). In the case of androstenone, *p*-values could not be obtained, since all values were under the detection limit of the equipment (0.20 µg/g). ^3^ bd: below the detection limit (0.20 µg/g). ^4^ Data were analyzed with non-parametric methods and are presented as mean ± standard deviation.

## Data Availability

Data available on request due to restrictions of privacy.
